# Atrial Fibrillation and Silent Coronary Spasm Complicating Severe Carbon Monoxide Poisoning

**DOI:** 10.7759/cureus.38768

**Published:** 2023-05-09

**Authors:** Ossma E ElBelihy, Amr Gebril, Ahmed E Abd Alhaleem, Tarek E Ibrahim, Ahmed B Osman, Hussein A Al Masalmeh

**Affiliations:** 1 Emergency Medicine, Madinat Zayed Hospital, Abu Dhabi, ARE; 2 Emergency Medicine, NMC Healthcare, Abu Dhabi, ARE; 3 Emergency Medicine, Yas Clinic Hospital, Abu Dhabi, ARE

**Keywords:** coronary spasm, oxygen therapy, atrial fibrillation, myocardial injury, carbon monoxide poisoning

## Abstract

Carbon monoxide (CO) poisoning is a toxicological emergency and may be responsible for more than half of all fatal poisonings worldwide. Serious effects of CO are frequently seen in the brain and heart as well as other organs that are particularly sensitive to hypoxia. Cardiac manifestations include dysrhythmias, myocardial infarction, and even cardiac arrest. Even in mild cases of CO poisoning with absent chest pain, the emergency physician should evaluate features of myocardial injury, as this can serve as a predictor of mortality and morbidity. A case of a young, healthy man with severe CO poisoning presented with atrial fibrillation (AF) and vasospastic angina, and he was managed successfully with high-flow oxygen.

## Introduction

Acute CO poisoning is a common poisoning-related emergency, and it is the leading cause of unintentional death in the United States. It may be responsible for more than half of all fatal poisonings worldwide [1,2].

Carbon monoxide poisoning has often been referred to as the silent killer [3], as it is a colorless, odorless, and tasteless gas produced by incomplete combustion of fuels or other materials containing carbon. It often goes unnoticed, results in severe toxicity, and usually affects many cohabitants. Its clinical presentation is non-specific, and the condition is frequently misdiagnosed [4].

The affinity of CO to hemoglobin is 250 to 300 times higher than that of oxygen, and carboxyhemoglobin (CO-Hb) is very stable and not easily dissociated. This can often result in hypoxia, especially in oxygen-sensitive organs [5]. Cardiovascular manifestations of acute CO poisoning include myocardial ischemia and infarction, dysrhythmias, and cardiac arrest [6,7].

A case of a young, healthy man with severe CO poisoning presented with atrial fibrillation (AF) and vasospastic angina, and he was managed successfully with high-flow oxygen.

## Case presentation

A 21-year-old male nonsmoker without a medical history of chronic illness was brought to the Emergency Medicine department in an unresponsive state after exposure to smoke while cooking with a gas cylinder in a closed room. The patient complained of headache, dizziness, and nausea after losing consciousness for a few minutes.

Upon examination, the patient had a clear, patent airway, with equal bilateral air entry and central trachea. His vital signs were a respiratory rate of 24 breaths/min, oxygen saturation of 99%, temperature of 36.8 °C, pulse rate of 133 beats/min with irregularities, blood pressure of 136/94 mmHg, and capillary refill time of less than two seconds. The patient was confused (Glasgow Coma Score of 13/15) with equally reactive pupils, no lateralization signs, no external signs of trauma, and a random blood sugar of 5.2 mmol/L.

**Figure 1:**
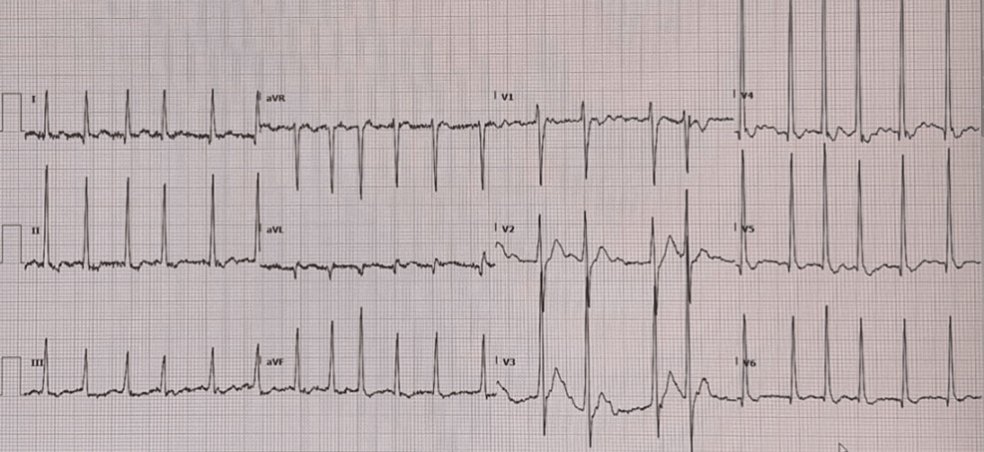
Initial ECG showing AF with a rate of 136 beats/minute ECG: electrocardiogram; AF: atrial fibrillation

An electrocardiogram (ECG) showed AF (Figure 1).

The CO-Hb level was 41.7% (normal: up to 3% in non-smokers and up to 10% in heavy smokers), pH was 7.373 (normal: 7.35 to 7.45), the pressure of carbon dioxide (PCO_2_) was 51.1 mmHg (normal: 35 to 45 mmHg), and bicarbonate (HCO_3_ ) was 26.5 mEq/L (normal: 22 to 26 mEq/L). The patient was diagnosed with severe CO poisoning and was administered high-flow oxygen with a non-rebreather mask, as no nearby hyperbaric oxygen chamber was available. Two hours into oxygen therapy, the patient became fully conscious and stopped experiencing headaches, dizziness, and nausea. Upon neurological examination, his cranial nerves II through XII showed to be intact without focal deficits.

Laboratory tests showed CO-Hb levels of 5.1%, so the patient continued oxygen therapy. Other vital statistics of note were troponin T, 14.3 ng/L (Normal: less than 14 ng/L); white blood cells, 8.19 x 10^9^/L (Normal: 4.5 to 11.0 x 10^9^/L); hemoglobin, 142.0 g/L (Normal: 138.0 to 172.0 g/L); platelets, 303 x 10^9^/L (Normal: 150 to 400 x 10^9^/L); sodium, 139 mmol/L (Normal: 136 to 145 mmol/L); potassium, 3.4 mmol/L (Normal: 3.6 to 5.1 mmol/L); creatinine, 73 µmole/L (Normal: 65.4 to 119.3 µmole/L); total creatine kinase, 185 U/L (Normal: up to 308 U/L); serum myoglobin, 28.3 µg/L (Normal: up to 72 µg/L); urine myoglobin, less than 21 µg/L (Normal: less than 21 µg/L); C-reactive protein, 0.7 mg/L (Normal: up to 5 mg/L); and international normalized ratio, 0.94 (Normal: 1.1 or below).

**Figure 2:**
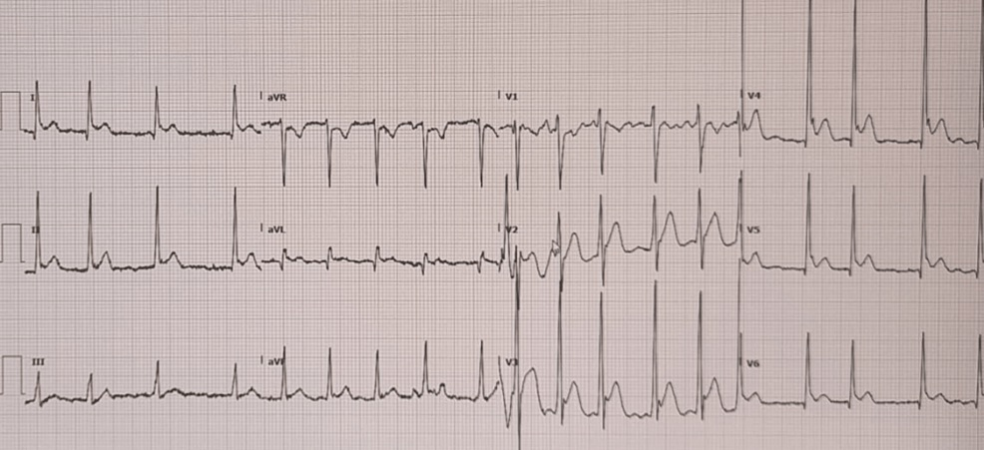
An ECG after two hours showing AF with a raised ST segment in pericardial leads ECG: electrocardiogram; AF: atrial fibrillation

After two hours, the ECG still showed AF with new ST-segment elevation in leads I, AVL, and V2 to V6 leads (Figure 2), and the patient had no dyspnea or chest pain. Aspirin 300 mg and clopidogrel 300 mg were administered, and an ST-elevation myocardial infarction code was announced. After 20 minutes, an ECG was repeated and showed AF, but the ST segment had returned to baseline. The cardiology team assessed the patient and suspected coronary spasm secondary to CO poisoning to be the cause of the transient ST-segment elevation. The patient was admitted to our facility and transthoracic echocardiography (ECHO) was performed and showed a preserved left ventricular systolic function with a left ventricle ejection fraction of 60% to 65%, no wall motion abnormalities, and non-significant trace mitral and tricuspid regurgitation with normal pulmonary artery systolic pressure. The patient was administered metoprolol 5 mg once daily and enoxaparin 40 mg daily. Hypokalaemia was corrected. Oxygen therapy was stopped once CO-Hb levels had dropped to a normal level of 1.2%. The ECG showed a return to normal sinus rhythm eight hours from the time of admission to the emergency. A coronary angiography was performed on the second day, showing normal coronaries. Serial troponin results came within the normal range. The patient was discharged after remaining in stable condition in inpatient observation for one day. The patient came for the outpatient follow-up after 10 days with no active complaints, normal vital signs, and a normal neurological exam. An ECG showed a normal sinus rhythm, and his follow-up ECHO study was normal.

## Discussion

The heart is among the organs most affected by CO poisoning because of its sensitivity to a lack of oxygen lack [8]. Acute CO toxicity can result in a wide range of negative effects on the cardiovascular system, including myocardial ischemia, infarction, dysrhythmia, cardiomyopathy, heart failure, cardiogenic shock, and sudden death [9]. The negative consequences of CO on the heart may result from tissue hypoxia due to increased CO-Hb formation as well as direct CO-mediated cell damage. Besides, CO has been reported to provoke coronary spasms, activate platelet aggregation, and initiate intracoronary thrombosis [10].

Acute CO poisoning increases the risk of developing dysrhythmias [11]. Sinus tachycardia, atrial or ventricular extrasystoles, AF, and atrioventricular conduction disorders (newly diagnosed bundle branch blocks) are frequently associated with CO poisoning.

Many mechanisms are involved in dysrhythmia development, including hypoxia-induced altered calcium gradients, increased diastolic intracellular calcium, increased calcium sensitivity of myofilaments, and a hyperadrenergic state [11]. Carbon monoxide increases nitric oxide levels, which leads to S-nitrosylation of the myocardial voltage-gated sodium channel. This increases the late component of the inward sodium current, which is pro-dysrhythmic [12].

The incidence of AF following CO exposure has been rarely reported. AF was documented in only 0.3% of cases from a cohort of 2,579 Korean patients who were diagnosed with acute CO poisoning [13]. Akdemir et al. described a case of acute CO poisoning in a 42-year-old woman who had experienced an episode of AF with a rapid ventricular response, which returned to normal sinus rhythm after receiving normobaric oxygen treatment for three hours. The researchers concluded that this short-term dysrhythmia was caused by a conduction disorder that resulted from tissue hypoxia [14]. Likewise, an AF rhythm following CO exposure in a 22-year-old male successfully reverted to normal sinus rhythm after treatment with hyperbaric oxygen [13].

A recent case report described a middle-aged woman who presented with non-radiating burning chest pain after soot inhalation. An ECG displayed progressive myocardial ischemia, as evidenced by progressive ST-segment elevation, accompanied by T wave inversion. Coronary angiographic examination revealed no obvious coronary stenosis, and the patient was diagnosed as a case of variant angina with coronary artery spasm induced by CO poisoning [15].

In our study, the patient had an ECG showing AF with transient ST-segment elevation and an increase in cardiac enzymes with normal coronary angiography. These findings were consistent with cardiac injury resulting from CO poisoning-induced coronary artery spasm and cardiac dysrhythmia. Earlier research work showed that myocardial injury can independently predict the short-term as well as the long-term negative consequences in cases of moderate-to-severe CO poisoning [7,8].

The extent of myocardial involvement and the clinical presentation of the case varies depending on the cardiovascular status of the patient. Even in individuals with minimal or no coronary atherosclerosis, severe CO toxicity is associated with acute coronary syndrome. Severe myocardial injury can also be seen with mild CO exposure in elderly patients who have cardiovascular risk factors [16]. Diagnosis is sometimes difficult, as typical chest pain can be missed in the early presentation [17] or it can be completely absent [18]. Haliga et al. found that dysrhythmia was more common with mild CO poisoning (17.2%) compared to levels of 7.7% and 8.7% in cases of moderate and severe poisoning, respectively [19].

Because of the urgency of the condition, even mild cases of CO poisoning and those with absent chest pain, the features of myocardial injury should be identified in every possible way, including screening with ECG, cardiac markers, as well as other laboratory tests (e.g., B-type natriuretic peptide) and imaging techniques (e.g., ECHO) [20].

## Conclusions

Correction of tissue hypoxia and reduction of CO-Hb half-life are major concerns in the management of CO poisoning. Normobaric or hyperbaric oxygen therapy should be used to improve the oxygenation status and enhance blood circulation to prevent thrombus formation and avoid injury to vital organs.
